# An Explainable Machine Learning Approach to Explain the Effects of Training and Match Load on Ultra-Short-Term Heart Rate Variability in Semi-Professional Basketball Players

**DOI:** 10.3390/s25226928

**Published:** 2025-11-13

**Authors:** Jorge Abruñedo-Lombardero, Alexis Padrón-Cabo, Daniel Vélez-Serrano, Alejandro Álvaro-Meca, Eliseo Iglesias-Soler

**Affiliations:** 1 Performance and Health Group, Department of Physical Education and Sport, Faculty of Sports Sciences and Physical Education, University of A Coruna, 15179 A Coruña, Spain; eliseo.iglesias.soler@udc.es; 2 Faculty of Education and Sport Sciences, University of Vigo, 36005 Pontevedra, Spain; apadron@uvigo.gal; 3 Department of Statistics and Operations Research, Universidad Complutense de Madrid, 28040 Madrid, Spain; 4 Department of Preventive Medicine and Public Health, Faculty of Health Sciences, Universidad Rey Juan Carlos, 28922 Madrid, Spain; 5 Networked Biomedical Research Center for Infectious Diseases, Instituto de Salud Carlos III, 28029 Madrid, Spain; 6 High-Performance and Innovation Research Group in Clinical and Community Epidemiology, Universidad Rey Juan Carlos, 28922 Madrid, Spain

**Keywords:** machine learning, SHAP analysis, heart rate variability, load monitoring, basketball

## Abstract

**Highlights:**

**What are the main findings?**

- Heart rate variability showed sensitivity to different measures of training and match load across the season.
- An individualized, explanatory modeling approach helped to identify which load variables influenced the internal response and in what direction.

**What is the implication of the main finding?**

- Monitoring heart rate variability alongside training load can inform athlete management strategies in team sports.
- The methodological framework highlights how individualized, explainable analyses can refine the dose–response process, even if further validation is needed in other contexts.

**Abstract:**

Understanding how training and match load influence autonomic recovery is essential for optimizing athlete monitoring. This proof-of-concept study aimed to examine the impact of training and match load on next-day heart rate variability (HRV) and to explain how different load measures influenced the internal response, using SHapley Additive Explanations (SHAP) to interpret machine learning models. Five semi-professional basketball players (23 ± 5 years; 191 ± 7 cm; 90 ± 11 kg) were monitored throughout a competitive season. HRV and load metrics were recorded daily. Differences in the natural logarithm of the root mean square of successive differences (LnRMSSD) across Non-Training, Training, and Match days were analyzed using linear mixed models. Additionally, a Gradient Boosting Machine model was developed to examine next-day HRV responses, with SHAP analysis providing both global and individual insights into feature importance. Next-morning LnRMSSD values were significantly lower on Match days compared to both Training and Non-Training days (*p* < 0.001). SHAP results identified rate of perceived exertion (RPE), days since last match, minutes played, and recent training load as the most influential variables associated with HRV changes. Pre-session heart rate and the root mean square of successive differences (RMSSD) values also demonstrated notable individual relevance. The ranking and magnitude of influential variables varied across players, highlighting the heterogeneity of physiological responses in team sports. While these findings are specific to this cohort, they illustrate the potential of explainable machine learning to enhance transparency and support individualized monitoring strategies. Importantly, they underscore the value of integrating both subjective and objective load measures to inform training decisions. Future research involving larger, multi-team samples is needed to validate the generalizability of these results.

## 1. Introduction

In team sports, where the competitive period spans several months, a well-structured approach to load quantification is essential [1,2]. During a competitive microcycle, sport scientists must monitor load and recovery to promote adaptation, minimize fatigue, and reduce injury risk in team-sports athletes [1,3]. Training load can be categorized into external and internal components, with external load referring to the work performed by the athlete during training or competition, and internal load referring primarily to the physiological response [2,4,5]. Basketball involves intermittent high-intensity actions and diverse player profiles across positions, each with distinct physical and tactical demands [6,7,8]. Combined with a congested schedule, this underscores the need for individual monitoring of internal and external load. A common method for load monitoring is heart rate (HR)-based measurements [9,10]. Metrics derived from HR monitoring, such as heart rate variability (HRV), can provide insights into the status of the autonomic nervous system and its potential relationship with fatigue [9,10]. HRV, sensitive to the prior day’s stimulus [4,11,12,13], can support assessment of physiological response and optimize the dose–response process throughout the competitive microcycle in basketball players.

HRV refers to the fluctuation in the time intervals between consecutive heartbeats [14]. Short-term HRV, particularly Root Mean Square of Successive Differences (RMSSD), reflects vagal modulation of cardiac autonomic activity and is sensitive to acute physiological stressors, such as high-intensity training or competition [9]. Reductions in RMSSD are typically associated with increased sympathetic activation and/or delayed parasympathetic recovery, whereas higher values indicate parasympathetic predominance and a more recovered state. It responds differently to high- and low-intensity stimuli [15], and exhibits individual variation [12,16,17], highlighting the need for individualized monitoring and analysis for each athlete. Among various HRV metrics, RMSSD and its logarithmic transformation (LnRMSSD) have been widely used for fatigue monitoring in team sports [11,12,16,18,19]. However, particular responses to load translates into interindividual differences in LnRMSSD responses to training [12,18], highlighting the athlete heterogeneity in team sports. On the other hand, HRV-based load modulation usually involves adjusting training based on the morning HRV reading [13], but this is often limited by time constraints in team settings. Therefore, explainable modeling of HRV responses to training loads could improve planning and decision-making during the competitive microcycle. Additionally, given the length of the competition period in team sports, considering HRV trends and load evolution across the entire season is also important. However, most studies have focused on partial periods such as pre-seasons or short cycles [11,17,18,20]. Likewise, there does not appear to be a clear consensus in the literature regarding the relationship between HRV and other load-related variables, as this relationship tends to be highly individual and vary depending on the monitoring method used [11,18,19,21,22]. Consequently, more research using explainable modeling approaches is needed to better characterize these complex relationships and enhance proactive load management in basketball players.

Traditional statistical models may face limitations in this context, as they often assume linear relationships and struggle to capture complex, non-linear, or interacting effects among multiple load variables, especially when these effects can vary substantially between individuals. These constraints can limit their ability to fully describe the multifactorial nature of training load–HRV interactions in team sports. In this context, advanced methods such as the SHapley Additive exPlanation (SHAP) technique offer a unique opportunity to enhance the interpretability of grey-box algorithms such as Gradient Boosting Models by identifying the contributing variables and enabling timely and informed adjustments [23]. This one can be used to relate a response variable, such as LnRMSSD with load variables that mostly influence on athlete’s fatigue reflected in HRV changes. SHAP technique provides values (SHAP values), which would allow us to know the contribution (positive or negative) of each load variable to the LnRMSSD estimation given by the model for each athlete and, globally, the importance and contribution of each variable load in the model. This is particularly valuable in team sports, where the interaction of multiple complex variables can complicate data-driven decision-making.

Therefore, the aims of this study were: (i) To assess the effect of the previous day’s stimulus on HRV; (ii) To build a model that explains which load variables affect HRV in basketball players, based on monitoring them throughout an entire season; (iii) To identify individual profiles regarding the specific factors that influence HRV changes; (iv) To demonstrate the utility of an explainable machine learning framework for providing actionable insights in athlete monitoring. We hypothesized that the training or match load from the previous day could influence the HRV response on the following day, and that specific load variables would explain this modification to a greater extent. Additionally, we expected to find individual differences among athletes in the factors influencing these responses.

## 2. Materials and Methods

### 2.1. Study Design

A longitudinal observational study with a repeated measures design was conducted, recording training load variables, rate of perceived exertion (RPE), training and match volume and HRV throughout a semi-professional basketball season in Spain (2021–2022). Daily recordings were performed, and only players who completed the full monitoring were included in the statistical analysis. Data were collected over a period of seven months (October to April) by the strength and conditioning coach as part of their professional development.

### 2.2. Participants

Five semi-professional basketball players (age: 23 ± 5 years; height: 191 ± 7 cm; body mass: 90 ± 11 kg) from a team competing in the Spanish EBA League (fourth division) voluntarily participated in this study. Based on playing positions, they were grouped as: 2 point guards, 2 wings, and 1 center. Each player contributed an average of 212.6 ± 23.9 HR and HRV recordings and 257 RPE and volume recordings. A slightly lower number of HRV recordings compared to RPE and volume data resulted from occasional missed or excluded measurements due to insufficient signal quality, as determined by the app’s validation protocol. The typical weekly schedule during the season included four training sessions and one match. On average, each player had 98 ± 9.77 training session recordings and 26.6 ± 2.19 match recordings. All participants were fully informed about the study procedures and gave their consent for the use of their data. Additionally, written informed consent was obtained from the club to analyze the data collected throughout the season. As data used in this study were collected as part of routine player monitoring, no ethics committee approval was required [24].

### 2.3. Procedures

#### 2.3.1. Training Days Classification (MD-TD-NTD)

**Figure 1:**
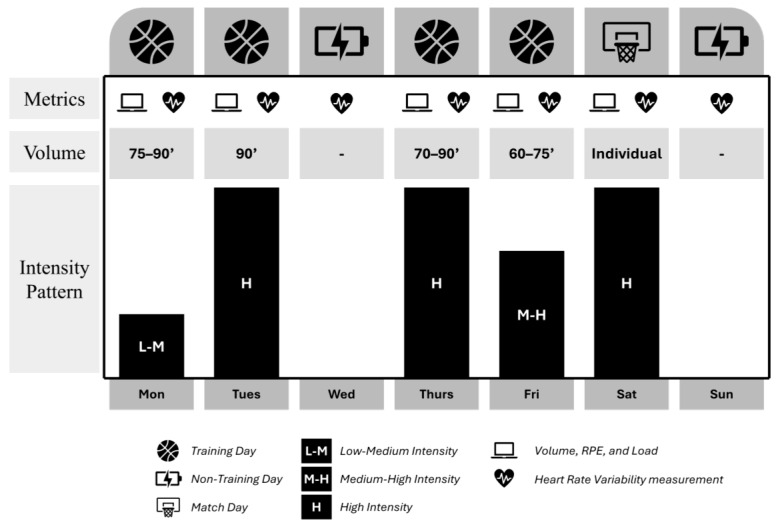
Microcycle type during season. Distribution of load during a microcycle and days where hear rate metric were recorded.

During the season, days were classified into three categories based on their content: match day (MD), training day (TD), or non-training day (NTD). A match day was defined as any day the player participated in at least one minute of an official game. A training day was recorded when there was a team training session in which the player participated fully. A non-training day was recorded when the player did not participate in either a game or training session that day. The typical in-season microcycle was structured as shown in Figure 1.

#### 2.3.2. Training Load

The primary load metrics recorded were training session duration or match minutes played, and subjective effort assessment via RPE. From these two metrics, session-RPE (sRPE) was calculated by multiplying volume and RPE. RPE is a valid tool for recording training load [25]. All metrics used can be found in Table A1. RPE data were collected using the TrainingFeel app (Atlántida Apps, Atlántida, Uruguay; https://trainingfeel.com/), which enables automated reporteing of Borg’s 1–10 scale by each player on their own mobile device. Data collection was preceded by a two-week familiarization phase and conducted individually. Each player answered the question “How intense did the session feel?” on their mobile device 15–30 min post-session. Session duration for each player was monitored by the team’s strength and conditioning coach, while match minutes were obtained from the official records on the Spanish Basketball Federation’s website (https://www.feb.es/, accessed on 15 July 2022).

For each of these variables, the moving average, weighted average, and exponentially weighted moving average (EWMA) were calculated [26]. A more detailed explanation of the EWMA can be found in Table A1. The average was calculated as the mean of the last few days, with the number of days included indicated in the variable name (e.g., sRPE_avg4). The weighted average was calculated by weighting the same average, giving a higher value to the most recent day (4 in the case of a four-day period) and reducing by one for each preceding day. The EWMA was calculated using the formula previously applied in other studies on load monitoring in sports [27].

#### 2.3.3. Heart Rate Variability

To monitor load responses, HR data were collected during training sessions and matches [10]. From these, HR, RMSSD, and LnRMSSD were derived. HRV monitoring, particularly through LnRMSSD, has been used by various authors as a method to assess the response to training load [9,16].

For HR data collection, participants downloaded the validated HRV4Training app [28] (HRV4Training, Amsterdam, The Netherlands; http://www.hrv4training.com/) and consented to data recording and processing for professional development and research purposes via the app itself. Each morning, upon waking, HRV was measured in a one-minute supine recording to avoid external stressors [29]. A measurement was considered valid if confirmed by the app’s algorithm. Ultrashort recordings (≤1 min) have shown validity and are sensitive to the prior day’s stimulus [30], with 1 min measurements, such as those used in this study, demonstrating good agreement with standard 5 min recordings while offering practical advantages for field-based monitoring, despite their lower precision compared to longer recordings [31]. The measurement was conducted using the photoplethysmography (PPG) method. PPG was used as a non-invasive and validated method to assess autonomic nervous system status [30], with participants placing their finger over the mobile device’s camera and flash. PPG is measured via reflection through the illumination of the skin using a light-emitting diode and through detection of the amount of light that is reflected by a photodetector or a camera located next to the light source [28]. The signal was acquired at 30 Hz through the smartphone camera [28]. The signal obtained was processed to isolate pulsatile blood flow, filtered to reduce noise, and up-sampled to allow accurate detection of inter-beat intervals and HRV calculation, as described in a previous validation study [28]. Data were uploaded automatically to a cloud server and accessed by the strength and conditioning coach.

### 2.4. Statistical Analyses

The linear mixed-effects model analysis was conducted to examine the effects of different types of days (MD, TD or NTD) on HRV, specifically LnRMSSD. The day category was set as a fixed effect factor, whereas the intercept was allowed to vary randomly across participants to account for individual differences in LnRMSSD for the reference condition (i.e., NTD). Post hoc contrast was performed by paired *t*-tests with Bonferroni’s correction. Normality of the residuals was visually inspected (i.e., histogram and Q-Q plots) whereas kurtosis and skewness statistics were also considered.

On the other hand, we aimed to adjust a model that allowed establishing the dependency relationships between the LnRMSSD variable and load indicators of basketball players. Although there are quite popular and interpretable techniques such as decision trees and regression models, the fact that the relationships involved in the current study can be especially complex led us to opt for more sophisticated models. Among them, we used an Extreme Gradient Boosting (XGBoost), which consists of the sequential adjustment of hundreds of decision trees, where each one assigns greater weight to the patterns worst predicted by the previous trees. This is why it is considered as a grey-box type model, whose results are competitive, from a predictive point of view. XGBoost was selected because it allows modeling potential non-linear and interacting effects among multiple training load variables without requiring explicit specification, offering flexibility and robustness for exploratory, proof-of-concept analyses in team sport settings. In this context, the SHAP technique is applied to quantify the contribution of each variable in the model (i.e., load variables; see Table A1) to the estimated outcome (LnRMSSD) through the computation of SHAP values. Furthermore, this interpretation can be performed both at a global level, to identify the most relevant variables to characterize the LnRMSSD, and at a local level, that is, at the athlete level. The latter is decisive to be able to make personalized decisions about each of them. Thus, one variable may be the one that generally contributes the most to an increase or decrease in the LnRMSSD, and yet another variable may be the one that is most relevant for a specific athlete. In this study, the reference level of LnRMSSD was defined as the average of NTD ± 0.5 SD within the last 30 days and therefore being updated daily.

**Figure 2:**
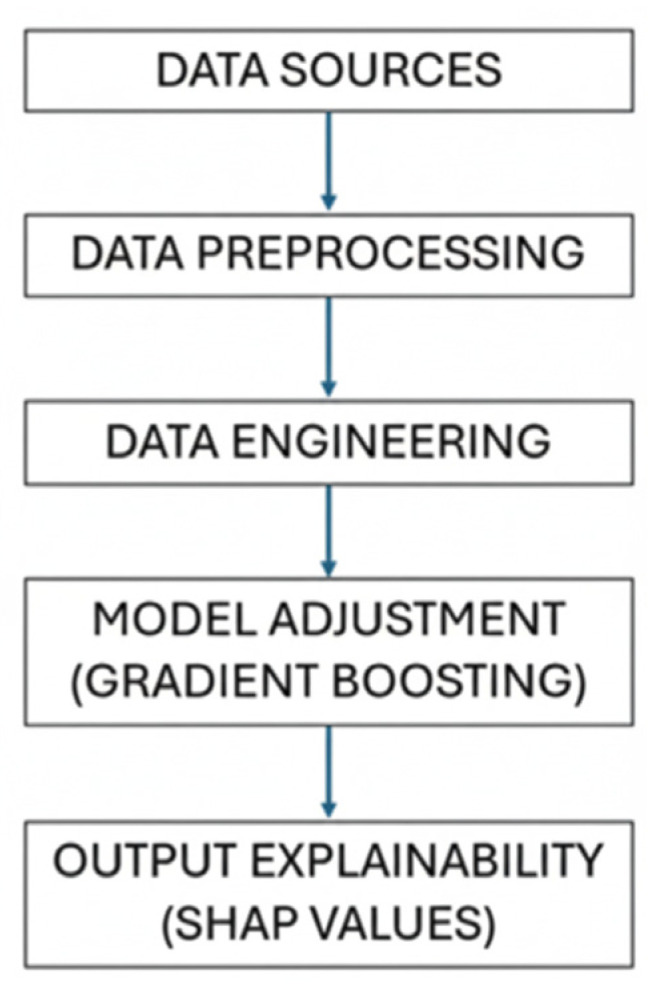
Schematic representation of the XGBoost modeling pipeline.

LnRMSSD was estimated using an Extreme Gradient Boosting Model. Regarding the model configuration, hyperparameters were tuned using grid search five-fold cross-validation minimizing the root mean squared error (RMSE). Hyperparameters were tuned using a grid search with five-fold cross-validation. The parameter grid included the following values: maximum tree depth (3, 4, 5, 6), learning rate (0.01, 0.05, 0.1), and number of trees (300, 400, 500). All other parameters were kept at their default values. Figure 2 illustrates the XGBoost modeling pipeline applied in this study, from data preprocessing to model explainability. Daily training load and HRV data were preprocessed, new indicators were engineered to capture acute and accumulated demands, and the model was trained to estimate next-day LnRMSSD. To obtain explanations of the features that drive players-dates estimates, we used a SHAP algorithm. SHAP plots provided a clear visualization of how each predictor contributed to the model’s estimation of day-to-day changes in LnRMSSD, allowing the interpretation of both the relative importance and the direction of their influence on the predicted response. The application of SHAP plots allowed us to analyze the impact of individual features on LnRMSSD estimates, encompassing both the direction and magnitude of their influence. In these plots, the magnitude of the value of the load variables is represented by colors, while the influence over the predicted given by the model is expressed by a Shapley value. In fact, these values represent the contribution (positive or negative) of each load variable to the LnRMSSD estimation given by the model.

By closely examining SHAP values, this analytical approach facilitated the identification of the input variables in the estimate of LnRMSSD. Thus, once a variable and an observation have been fixed, it will be represented by a dot. This dot will be associated with a more intense red color, the higher the value of the variable on that observation, and a more intense blue color the lower the value. In addition, the higher the Shapley value (i.e., the farther to the right the observation is represented), the greater the contribution of the variable to the increase in the estimate given by the model for that observation. Conversely, the lower the Shapley value (i.e., the further to the left the observation is represented), the greater the contribution of the variable to the decrease in the estimate given by the model for that observation. In consequence, the sign informs whether the variable contributes positively or negatively to the estimate made for the observation. According to their global influence on the model, the variables will be presented from greater to lesser importance on the *Y* axis.

On the other hand, linear mixed model analysis was carried out using SPSS software v.28 (IBM Corp., Armonk, NY, USA), with a significance level set at *p* < 0.05, whereas the explanatory framework model was performed using the Python package XGBoost in Python 3.11.3 (Python Software Foundation, Beaverton, OR, USA)

## 3. Results

Values of LnRMSSD were analyzed according to the categorization of days using mixed models. The estimated marginal means for each type of day were: 4.711 ± 0.146 ln ms for NTD, 4.653 ± 0.146 ln ms for TD, and 4.408 ± 0.149 ln ms for MD.

**Figure 3:**
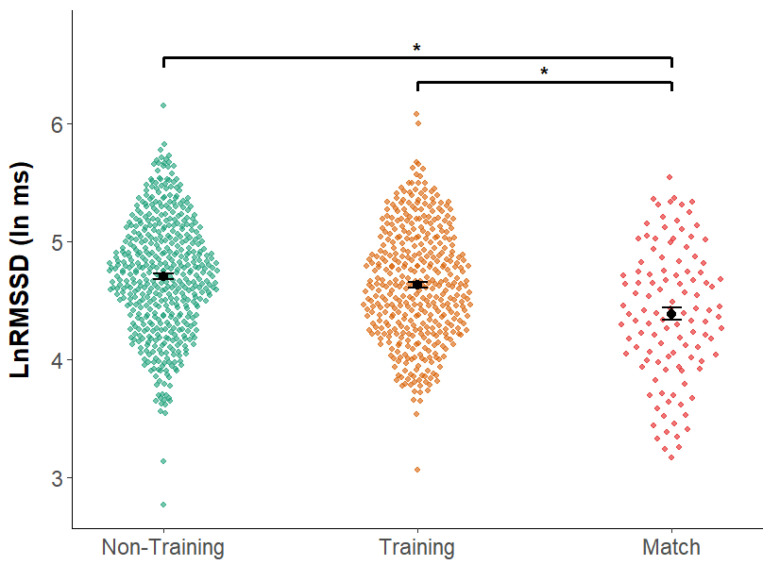
Distribution of daily LnRMSSD (ln ms) by day type: Non-Training Day (NTD), Training Day (TD), and Match Day (MD). Each dot represents one daily observation (jittered for visibility) across players. Black points with horizontal error bars show estimated means ± SE for each day type. Horizontal brackets denote pairwise differences from a linear mixed-effects model with subject as a random intercept. * Significant difference at *p* < 0.05.

Pairwise comparisons (Figure 3) revealed significant differences between NTD and MD (mean difference = 0.304, *p* < 0.001) and between TD and MD (mean difference = 0.245, *p* < 0.001), whereas non-significant differences were observed between NTD and TD (mean difference = 0.058, *p* = 0.578).

The predictive performance of the XGBoost model was evaluated using five-fold cross-validation. The model achieved a cross-validated RMSE of 0.2683 ± 0.0126 and a training RMSE of 0.2567, suggesting consistent performance between training and validation and supporting the stability of the model fit. Although the primary objective of this study was explanatory rather than predictive, reporting these values supports the robustness of the model in characterizing the relationships between training load variables and LnRMSSD.

**Figure 4:**
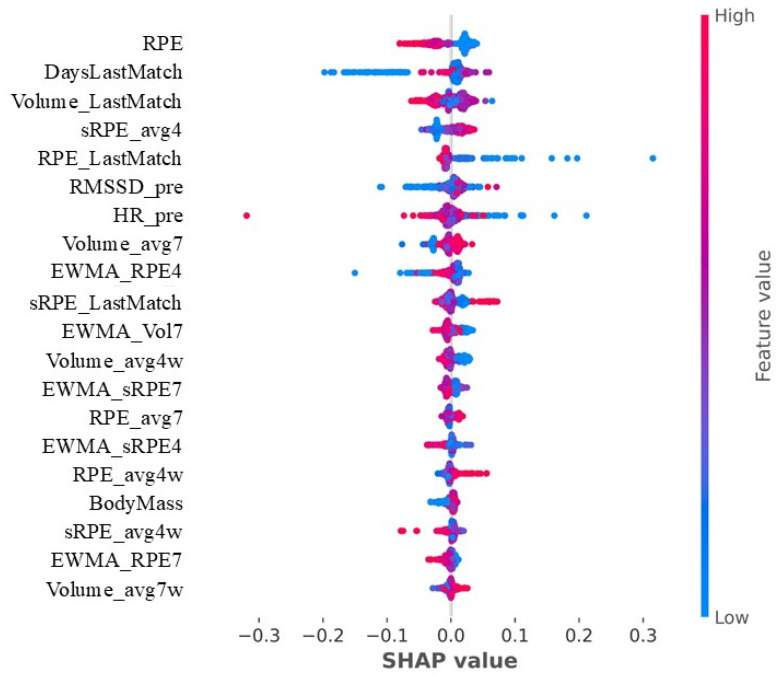
Most relevant variables for characterizing LnRMSSD. The order indicates their importance in modifying LnRMSSD. The position on the *x*-axis (SHAP value) indicates the direction and magnitude of each variable’s impact on the model’s output. Features on the left side of the vertical line (SHAP value < 0) are associated with a decrease in LnRMSSD, while features on the right (SHAP value > 0) are associated with an increase. The color gradient represents the feature value, with red indicating higher and blue indicating lower values. The description of each abbreviation is provided in Table A1.

The SHAP analysis shows the contribution of different variables to the modification of LnRMSSD (Figure 4). The most impactful variables include RPE, the number of days since the last match (DaysLastMatch), minutes played in the last match (Volume_LastMatch), the average sRPE of the last four days, and the RPE of the last match (RPE_LastMatch), among others. Additionally, variables such as pre-training or pre-match RMSSD and HR also influence the modification of LnRMSSD on the following day.

**Figure 5:**
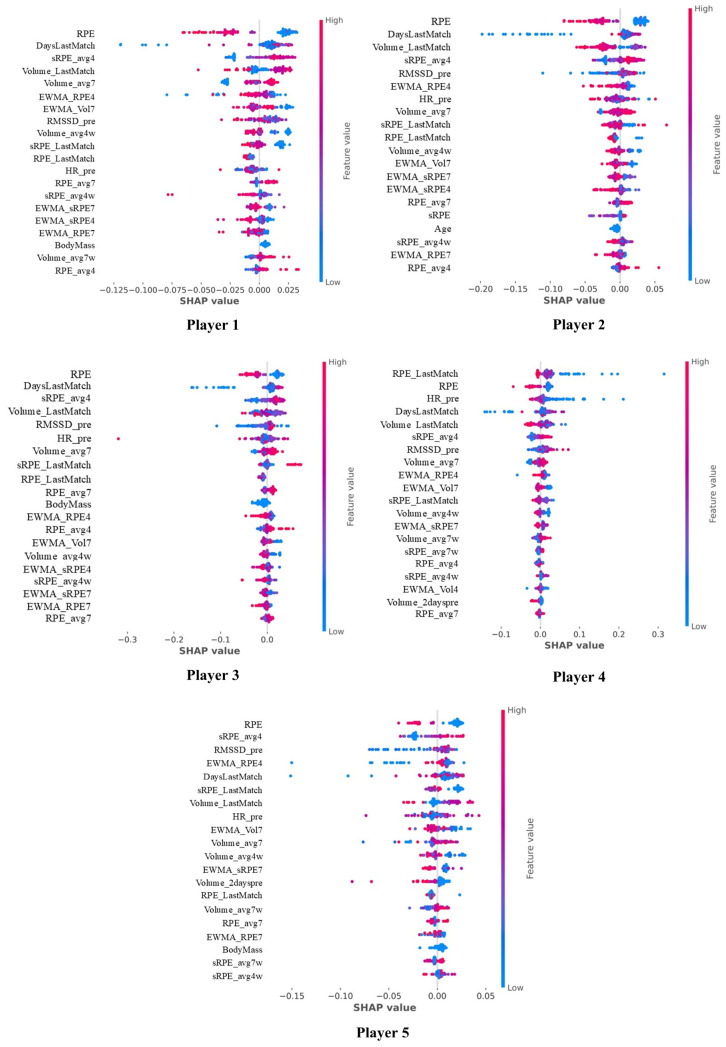
Most relevant variables for characterizing LnRMSSD for each player. The direction of the SHAP value (positive or negative) indicates the direction of each variable’s effect relative to the model’s prediction for LnRMSSD. The description of each abbreviation is provided in Table A1.

Figure 5 displays the individual analyses, highlighting inter-individual differences in the order, weight, and impact of the variables associated with HRV changes for each player. To further illustrate how training load variables interact and jointly influence next-day LnRMSSD, SHAP interaction dependence plots were generated and are provided as Supplementary Material (Figures S1 and S2).

## 4. Discussion

The main findings of this study are: (i) LnRMSSD is influenced by the training load from the previous day, with matches being the stimulus that induces the greatest change; (ii) intensity metrics appear to be one of the most important variables explaining LnRMSSD variation on the following day; (iii) individual differences exist regarding which variables most influence LnRMSSD modification in each player.

Previous research has shown that HRV is influenced by training load in team-sport athletes [12,19]. Consistent with these findings, our results indicate that LnRMSSD is affected by training load in semiprofessional basketball players. However, those studies focused on short-term periods [11,12,18] such as a single competitive microcycle or preseason. In contrast, the present study monitored players across a full competitive season. Specifically, our results show a significant difference between MD and both TD and NTD, while no differences were found between TD and NTD. This may be due to the variability of training sessions, which ranged from low to high intensity. Nakamura et al. [32] reported a decrease in LnRMSSD the morning following matches during a beach volleyball tournament, though they did not compare this response to training sessions, leaving uncertainty about HRV responses to different stimuli. Our findings appear to support structuring the microcycle around the match and its proximity, as the match appears to elicit the largest suppression of LnRMSSD. One of the limitations of previous studies is the lack of direct comparisons between physiological responses to matches and training. Future research should address this to determine whether matches consistently cause greater HRV reductions and to clarify the implications of this response.

Through SHAP analysis, RPE and days since the last match were identified as the two most influential variables in LnRMSSD changes. Consistent with previous research [15,30], perceived intensity through RPE appears to be the main factor influencing HRV [33]. In a previous study, O’Connor et al. [19] reported a relationship between sprint volume and a reduction in RMSSD upon waking the following day. Similarly, Stanley et al. [15], found that while cardiac autonomic recovery tends to recover within 24 h following low-intensity exercise, high-intensity sessions suppress it for up to 48 h post-exercise. Our findings, highlighting RPE as the main variable, align with these authors, reinforcing the impact of exercise intensity on next-day LnRMSSD suppression. From an applied perspective, this finding supports the use of RPE as a simple and low-cost monitoring tool to track internal load and anticipate short-term changes in players’ physiological state. Although HRV-derived parameters such as LnRMSSD provide valuable information on autonomic modulation, the practicality and immediacy of RPE make it especially useful for daily load management in team sport environments. The number of days since the last match ranks second, suggesting that having a match the previous day negatively affects LnRMSSD, which highlights the importance of organizing the days following the match due to their high-intensity nature, and the need for at least 48 h for full parasympathetic cardiac reactivation [15]. These findings suggest that match intensity and recovery window should be key considerations in basketball microcycle planning. Minutes played in the match also significantly influenced LnRMSSD, with higher values of minutes played linked to decreased LnRMSSD the following day. We also identified a third key metric related to the match: the reported RPE metric that refers to the RPE reported in the last match played. Therefore, these results seem to highlight the importance of the match in LnRMSSD response, along with minutes played and match RPE as a key metrics to anticipate players’ LnRMSSD changes. Since HRV can also be influenced by psychological variables [34], match demands and outcomes may play a role in the magnitude of these changes. The average sRPE of the last 4 days showed an inverse relationship with the next-day LnRMSSD. Since it links both volume and perceived intensity metrics, the response may be primarily influenced by the volume, as it takes higher values than RPE. This effect was clearer when analyzing the 7-day average volume, which appeared to positively influence LnRMSSD. This suggests that training exposure may help sustain favorable LnRMSSD values [21], although acute responses depend on session intensity [15,19]. Pre-stimulus variables such as HR and pre-match RMSSD also play a significant role in the response the following day. Including previous-day RMSSD in the model allowed us to consider the autoregressive nature of HRV, acknowledging that the athlete’s prior autonomic state influences next-day recovery and response. This interpretation aligns with previous evidence describing how parasympathetic reactivation and day-to-day HRV fluctuations reflect the dynamic balance between stress and recovery [9,15]. Higher resting HR or lower RMSSD values prior to training seem to influence a greater reduction in LnRMSSD the next day. Taken together, these results suggest the need to plan training loads during the microcycle by taking into account not only the match and the intensity of training sessions, but also the athlete’s physiological status prior to each session. Considering the clear impact that match-related variables and perceived intensity have on HRV modulation, practitioners should incorporate these findings into the weekly planning process to optimize the balance between stimulus and recovery and ultimately support performance and adaptation.

Individual differences were observed in the training load metrics associated with LnRMSSD responses. Similarly, Flatt et al. [16] reported in American football, differences among players. This highlights the importance of individualizing the monitoring process based on the specific characteristics of each athlete. Each player exhibits a unique profile in which specific metrics have varying degrees of importance and magnitude in LnRMSSD modification. To better illustrate these individual differences, Supplementary Figure S3 presents a heatmap summarizing the relative importance and ranking of key training load variables for each player. The individualized SHAP analysis revealed distinct physiological and load-response profiles among players, confirming that the factors influencing LnRMSSD are not homogeneous across the team. A clear contrast emerged between players whose autonomic response was driven primarily by external or perceived load and those with a stronger physiological dependence on pre-session states. For example, Players 1 and 4 were predominantly influenced by RPE and match-related variables, indicating a heightened sensitivity to perceived intensity and competitive stress. In contrast, Player 5 showed a more physiologically driven profile, with RMSSD_pre among the most influential variables, suggesting that his baseline autonomic state before training or competition plays a decisive role in modulating next-day LnRMSSD. A second pattern distinguished players whose response was shaped mainly by acute versus accumulated load. While RPE and DaysLastMatch were consistent markers across the group, Player 5 showed a notable influence of recent training volume (sRPE_avg4), pointing toward a greater impact of short-term load accumulation on his autonomic recovery. These contrasting profiles illustrate that while certain indicators (e.g., RPE, match proximity) are relevant at a team level, their relative weight varies substantially between individuals. From a practical standpoint, this supports differentiated monitoring strategies: for players more dependent on their physiological baseline (e.g., Player 5), daily HRV assessments can help tailor session intensity; for those more sensitive to perceived or match-related load (e.g., Players 1 and 4), managing post-match recovery windows and controlling intensity may be more critical. Players with stronger responses to accumulated load may benefit from progressive load modulation within the microcycle. Altogether, these findings highlight the added value of explainable models to move from uniform to player-specific monitoring frameworks, allowing practitioners to better align training and recovery strategies with the individual load–response profile of each athlete.

To the best of the authors’ knowledge, this is the first study to apply SHAP analysis to model athlete fatigue, considering different types of variables recorded throughout an entire competitive season. This technique enables the development of both general and individual explanatory models, rather than relying on the retrospective decision-making approach provided by other analytical methods. Our results showed that RPE and the number of days since the last match emerged as the main factors explaining variations in player condition, as assessed through LnRMSSD. However, distinct individual profiles were also identified. Machine learning techniques, combined with interpretability methods such as SHAP, offer a methodological framework to clarify how training loads relate to fatigue in team sports, allowing for the anticipation of training load adjustments.

There are some limitations in the present study that should be acknowledged. Firstly, the sample size represents the primary limitation as it restricts the ability to draw broader conclusions and limits the generalizability of the findings. Nevertheless, a substantial amount of data per player was used to train the mathematical model. Secondly, we analyzed only a single season, whereas a larger longitudinal study would be beneficial to confirm the findings. Nevertheless, to our knowledge, this is the first study to analyze data from an entire competitive season using explainable machine learning methods to provide insights into fatigue in team sports. Additionally, training load was assessed using RPE, which, although a valid and widely used measure of internal load, is subjective and may be influenced by factors such as mood and motivation. Moreover, psychological stress, which is known to affect HRV, was not measured and could represent a potential confounding variable, particularly around matches. In addition, the lack of external load measures (e.g., GPS, accelerometry) prevented direct comparisons between internal and external load drivers, which would be valuable for future research. Nevertheless, RPE and sRPE have been shown to reflect internal load in team sports [8,35]. Finally, although HRV4Training has been validated against ECG for short-term HRV analysis, PPG-based measurements can be more susceptible to artifacts and environmental factors compared to ECG. This should be considered when interpreting the precision and robustness of the results.

## 5. Conclusions

The results of the present study showed that LnRMSSD is significantly influenced by the training load from the previous day, with matches being the stimulus that induces the greatest change. RPE and the number of days since the last match, were identified as the main factors affecting LnRMSSD modification. Additionally, playing time in the last match and the physiological state before training or competition (RMSSD and HR) also influenced LnRMSSD responses on the following day. In addition, the individual analyses revealed differences among players regarding the most influential variables and their impact on HRV modulation. These findings highlight the importance of considering individual profiles when managing training load and monitoring fatigue status in basketball players.

To the best of the authors’ knowledge, this proof-of-concept study is the first to apply SHAP to explain machine-learning models of HRV-based athlete fatigue, as measured by LnRMSSD, over an entire competitive season in a team sport, enabling the identification of both general and individual response patterns to training load. The integration of machine learning techniques with interpretable methods such as SHAP provides a potentially valuable framework for anticipating training load adjustments and informing strategies aimed at optimizing performance and recovery in team sports, while recognizing that further studies are needed to validate its generalizability to other teams and contexts.

## Data Availability

The anonymized dataset generated and analyzed during the current study will be deposited in the institutional Zenodo community repository of the University of A Coruna and made publicly available upon publication.

## Supplementary Materials

The following supporting information can be downloaded at: https://www.mdpi.com/article/10.3390/s25226928/s1, Figure S1. SHAP dependence plot showing the relationship between *RPE* and *LnRMSSD*, with color coding representing *DaysLastMatch*; Figure S2. SHAP dependence plot showing the relationship between *DaysLastMatch* and *LnRMSSD*, with color coding representing *Volume_LastMatch*; Figure S3. op SHAP feature importance by player. Heatmap illustrating the most influential variables for each player, based on SHAP ranking (1st–5th position).

## Abbreviations

The following abbreviations are used in this manuscript:

EWMA	Exponentially Weighted Moving Average
HR	Heart Rate
HRV	Heart Rate Variability
LnRMSSD	Natural Logarithm of the Root Mean Square of Successive Differences
MD	Match Day
NTD	Non-Training Day
PPG	Photoplethysmograph
RMSE	Root Mean Squared Error
RMSSD	Root Mean Square of Successive Differences
RPE	Rate of Perceived Exertion
SHAP	SHapley Additive exPlanation
sRPE	Session Rate of Perceived Exertion
TD	Training Day
XGBoost	Extreme Gradient Boosting

## Appendix A

**Table A1:** List of variables included in the SHAP analysis.

Name	Definition
Height	Height in centimeters.
Age	Age at the start of the season
BodyMass	Body mass in kilograms
MD-TD-NTD	Classification of days based on whether it was a match day (MD), training day (TD), or non-training/non-match day (NTD).
Volume	Volume in minutes of session duration in the case of training days. For matches, the number of minutes played in the game.
Volume_2dayspre	Volume metric (in minutes) from the two days prior to the HRV measurement (as HRV is measured the morning following the stimulus).
Volume_avg4	Mean volume (in minutes) over the last 4 days
Volume_avg7	Mean volume (in minutes) over the last 7 days
Volume_avg4w	Weighted average of the volume over the last 4 days, with a weight of 4 for the most recent day, 3 for the following day, 2 for the next, and 1 for the day furthest back.
Volume_avg7w	Weighted average of the volume over the last 7 days, with a weight of 7 for the most recent day and decreasing by one each day until assigning a weight of 1 to the day furthest back.
Volume_LastMatch	Minutes played in the last match
EWMA	Exponentially Weighted Moving Average Method for monitoring load through the acute-to-chronic ratio, which assigns a decreasing weight to each older load value, thereby giving greater weight to recent load. EWMA = Metric × λ + ((1 − λ) × (EWMAyesterday)), Where Metric refers to the observed value in the load metric (RPE, Volume, etc.), Lambda represents a constant between 0 and 1 that determines the depth of how many days influence the calculation. Assigning a lower value means that older values retain significant weight for a longer period. EWMA yesterday refers to the EWMA value for the previous day.
EWMA_RPE4	EWMA of the RPE variable where lambda equals 4
EWMA_RPE7	EWMA of the RPE variable where lambda equals 7
EWMA_sRPE4	EWMA of the sRPE variable where lambda equals 4
EWMA_sRPE7	EWMA of the sRPE variable where lambda equals 7
EWMA_Vol4	EWMA of the Volume variable where lambda equals 4
EWMA_Vol7	EWMA of the Volume variable where lambda equals 7
HR	Heart rate (in beats per minute) on the day following to the stimulus.
HR_pre	Early morning heart rate (in beats per minute) on the same day as the training or match.
RMSSD	Calculating each successive time difference between heartbeats in milliseconds. Then, each of the values is squared and the result is averaged before the square root of the total is obtained.
RMSSD_pre	Early morning RMSSD in milliseconds on the same day as the training or match.
LnRMSSD	A natural log is applied to the RMSSD to smooth the data and facilitate interpretation.
RPE	Subjective rating of session intensity was assessed using Borg’s 1–10 scale, in response to the question, ‘How intense did the session feel?’
RPE_2days	RPE metric from the two days prior to the HRV measurement (as HRV is measured the morning following the stimulus).
RPE_avg4	Mean RPE over the last 4 days
RPE_avg7	Mean RPE over the last 7 days
RPE_avg4w	Weighted average of the RPE over the last 4 days, with a weight of 4 for the most recent day, 3 for the following day, 2 for the next, and 1 for the day furthest back.
RPE_avg7w	Weighted average of the RPE over the last 7 days, with a weight of 7 for the most recent day and decreasing by one each day until assigning a weight of 1 to the day furthest back.
RPE_LastMatch	RPE reported in the last match
sRPE	Result of multiplying the session volume in minutes by the RPE.
sRPE_2days	sRPE from two days prior (as HRV is measured the morning following the stimulus)
sRPE_avg4	Mean sRPE over the last 4 days
sRPE_avg7	Mean sRPE over the last 7 days
sRPE_avg4w	Weighted average of the sRPE over the last 4 days, with a weight of 4 for the most recent day, 3 for the following day, 2 for the next, and 1 for the day furthest back.
sRPE_avg7w	Weighted average of the sRPE over the last 7 days, with a weight of 7 for the most recent day and decreasing by one each day until assigning a weight of 1 to the day furthest back.
sRPE_LastMatch	Result of multiplying the minutes played in the last match by the RPE.
DaysLastMatch	Number of days since the player’s last match.
